# Analysis of the application of 3D‐printed personalized positioning fixation in nasopharyngeal carcinoma radiotherapy

**DOI:** 10.1002/acm2.70337

**Published:** 2025-11-05

**Authors:** Zeyu Ding, Guo‐quan Li, Qi Liu, Xiaohui Zhu, Lin Tan, Jun Kang, Ye Wang, Zhiyong Yang, Jinghua Ren, Jun Han, Pinjing Cheng

**Affiliations:** ^1^ School of Nuclear Science and Technology University of South China Hengyang Hunan Province China; ^2^ Cancer Center, Union Hospital, Tongji Medical College, Huazhong University of Science and Technology Wuhan Hubei Province China; ^3^ Sichuan Provincial People's Hospital Chengdu Sichuan Province China

**Keywords:** 3D printing, cone‐beam CT (CBCT), dose distribution, nasopharyngeal carcinoma, personalized immobilization, planning target volume (PTV), setup accuracy

## Abstract

**Objective:**

This study aims to evaluate the impact of personalized 3D‐printed headrest devices on setup errors and dosimetry in nasopharyngeal carcinoma radiotherapy.

**Methods:**

A total of 50 nasopharyngeal carcinoma patients (28 male, 22 female, aged 24–76 years, mean age 45 years) who received radiotherapy at our center between July 1, 2023, and August 10, 2024, were randomly divided into two groups. Group A: 3D‐printed headrest and thermoplastic mask (Klarity Medical); Group B: Standard headrest and thermoplastic mask. All patients underwent a simulation CT (Philips IntelliSpace; 120 kV/200 mAs, 3 mm slices) in a supine position. The VMAT plan (70 Gy/33 fx) was designed in Eclipse 15.6: Plan‐P: Actual PLA CT values included; Plan‐0: PLA CT values set to ‐1000 (control). Daily CBCT verification (Varian Halcyon) was performed to measure setup errors at the Clivus, C4, and C7 vertebral levels. The Van Herk formula was used to determine the appropriate planning target volume (PTV) margin. Dose measurements used PCI, HI, CR, Dmean, and Dmax to assess PTVs (GTVnx+nd, PTV1, PTV2) and OARs (brainstem, spinal cord, lens, optic nerve).

**Results:**

A total of 340 CBCT images were collected from 50 patients, with 164 images from Group A and 176 from Group B. Setup errors in Group B were generally larger than those in Group A. Statistically significant differences were observed in the AP direction at the Clivus, C4, and C7 vertebral ROI registrations. Roll rotational errors showed statistically significant differences in ROI registration at C4 and C7 vertebral levels. The external radiation margins in all directions for Group A were smaller than those for Group B, with the largest difference observed at the C7 vertebral level. The external margins for the C7 ROI registration in the LR, SI, and AP directions were 2.91, 2.97, and 3.01 mm, respectively. Compared with Plan‐0, the Dmean and CR of the target volumes PTVnd+nx and PTV1 showed statistically significant differences (*p* < 0.05). Differences in Dmean, CR, and PCI for PTV2 were also statistically significant (*p* < 0.05). For the dosimetric evaluation of critical organs adjacent to the target volume, statistically significant differences were observed in the maximum doses to the brainstem, spinal cord, left lens, left optic nerve, and right optic nerve between the two planning approaches (*p* < 0.05). However, no statistically significant differences were found in the mean doses to these organs at risk (OARs) (*p* > 0.05).

**Conclusion:**

The application of 3D‐printed immobilization technology significantly improves setup accuracy in nasopharyngeal carcinoma radiotherapy and reduces cervical spine displacement. The incorporation of 3D‐printed materials exerts a measurable influence on target volume dose distribution and may notably increase the maximum dose delivered to adjacent critical organs.

## 1 INTRODUCTION

Nasopharyngeal carcinoma (NPC) is a malignant tumor originating from the nasopharyngeal epithelium, with a high incidence in populations of South China and Southeast Asia.^1^ Intensity‐modulated radiation therapy (IMRT) is the primary treatment for early‐stage NPC, while combined chemotherapy is used for advanced stages.^2^, ^3^ IMRT achieves high‐precision irradiation of the tumor target area and adequate protection of adjacent critical organs by dose sculpting, resulting in a 5‐ and 10‐year local control rate of over 90% for early‐stage NPC patients.^4^, ^5^, ^6^, ^7^, ^8^ However, due to the deep anatomical location of the nasopharynx and its proximity to critical organs such as the brainstem, spinal cord, and optic nerves, radiation therapy alone may cause a series of acute and late adverse effects, including radiodermatitis, mucositis, dysphagia, hearing loss, and xerostomia.^9^ Accurate target area design can reduce the adverse effects caused by excessive radiation exposure.^10^ Even minor deviations in patient positioning during treatment may result in insufficient doses to the target area or excessive radiation to critical organs. Therefore, new technologies demand higher standards for positioning fixation techniques.

The random motion errors of the mandible and lower cervical vertebrae are considered one of the significant challenges in achieving precise fixation in head and neck radiation therapy.^11^ Although neck muscle training helps reduce positioning errors, the design of existing fixation molds still fails to sufficiently adapt to individual anatomical differences, resulting in approximately a 1% chance of errors exceeding 3 mm in all directions during treatment.^12^ The Alpha Cradle (AC) foam system, which expands to conform to the neck and shoulder anatomy, has shown excellent immobilization performance in NPC radiotherapy.^13^ However, during the process of generating solid foam, this material produces heat. It releases irritating gases, and excessive expansion may exert non‐physiological straightening forces on the neck, presenting complexities for its clinical application. Therefore, developing a new fixation method that is both highly adaptable to individual anatomical structures and easy to operate is crucial for enhancing the precision of radiotherapy for nasopharyngeal carcinoma.

In recent years, the availability and utilization of 3D printing technology in radiation therapy have increased significantly. It has been widely applied in external beam radiotherapy (EBRT) (e.g., personalized tissue compensators, phantoms, and fixation devices) and brachytherapy (e.g., afterloading therapy and particle implantation).^14^, ^15^, ^16^, ^17^ Researchers can utilize CT‐ or MRI‐based three‐dimensional reconstruction and additive manufacturing techniques to create personalized fixation devices that accurately conform to the anatomical structures of the head, neck, and shoulders.^18^ However, the application of this technology in radiotherapy positioning fixation is still in the exploratory stage, especially in the clinical treatment of tumors with complex anatomical structures, such as nasopharyngeal carcinoma. Polylactic acid (PLA), a commonly used 3D printing material^19^ has not been systematically studied for its dosimetric properties in the treatment of nasopharyngeal carcinoma with radiotherapy. This study aims to systematically evaluate the application value of 3D printing fixation technology in nasopharyngeal carcinoma radiotherapy, focusing on its impact on the positioning accuracy of the Clivus area, the fourth cervical vertebra (C4), and the seventh cervical vertebra (C7), as well as quantifying its role in optimizing the external beam boundary of the planning target volume (PTV). Additionally, for PLA material, this study will evaluate the impact of head and neck support molds made from PLA on the dose distribution of radiotherapy plans for nasopharyngeal carcinoma, providing evidence‐based support for optimizing clinical practice.

## 2 OBJECTS AND METHODS

### 2.1 Patients selection

A total of 50 patients with nasopharyngeal carcinoma who underwent radiotherapy at our center between July 1, 2023, and August 10, 2024, were randomly selected, with ages ranging from 24 to 76 years and a median age of 45 years. Among them, 28 were male (56%) and 22 were female (44%). The patients were divided into two groups: 25 cases in the 3D‐printed head and neck support fixation group (14 males and 11 females) and 25 cases in the standard headrest fixation group (14 males and 11 females). There were no statistically significant differences in age or gender between the two groups (*p* > 0.05). The inclusion criteria included patients with primary nasopharyngeal carcinoma, whose radiotherapy fields encompassed the primary tumor and lymphatic drainage regions. Exclusion criteria included patients who had received chemotherapy or radiotherapy at other institutions. Volumetric modulated arc therapy (VMAT) plans were developed for both groups, with a prescribed dose of 70 Gy delivered in 33 fractions, five sessions per week.

### 2.2 3D printing mold production

**FIGURE 1:**
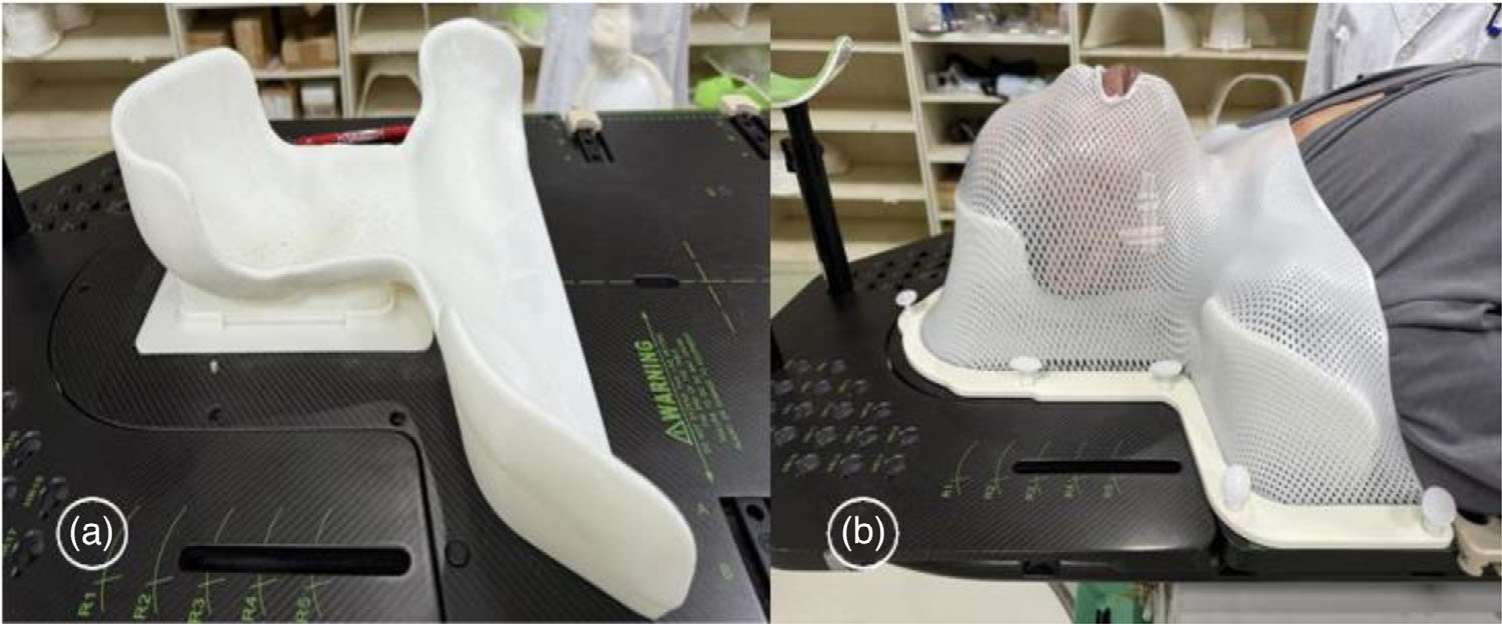
(a) 3D‐printed headrest support mold. (b) Patient positioning fixation with a combination of 3D‐printed headrest support and thermoplastic mask.

Patients in the 3D‐printed headrest support fixation group were positioned supine on the integrated positioning frame, with the chin raised at an appropriate angle, ensuring alignment of the midline of the frame, the patient's midline, and the laser positioning line. Subsequently, CT imaging was performed using parameters of 200 mAs, 120 kV, and a slice thickness of 1.5 mm, covering the area from the cranial vertex to the subclavian region. The imaging data were then transferred to the 3D printing system. Based on the CT image data, a personalized head‐neck‐shoulder support model was constructed (Figure 1a) using 3D printing. A high‐precision standard (2%/1 mm) was adopted, and the material thickness was controlled within ≤4 cm. The Hongrui Z600Plus 3D printer was employed to fabricate the headrests, utilizing a heated print head and a 0.4‐mm nozzle to extrude polylactic acid (PLA) and form both support and solid structures. Each 3D‐printed headrest featured an external shell with a thickness of 0.8 mm and an internal 2D infill composed of 10% PLA, with an overall thickness not exceeding 4 cm. Experimental measurements confirmed a radiation transmission rate greater than 97%. The material demonstrated both biocompatibility and patient comfort, meeting clinical usability requirements. Following fabrication, the surfaces were polished to achieve smoothness, and once they satisfied clinical standards, the headrests were delivered to the radiation therapy center for clinical application.

### 2.3 Computed tomography and patient immobilization

The same experienced radiation therapist immobilized all cases. The immobilization procedures were carried out according to the recommended protocols of the Halcyon (Varian Medical Systems, Palo Alto, CA, USA) treatment system. Patients were positioned in the supine position, with their bodies relaxed and both arms placed alongside their thighs. The optimal positioning was based on the patient's comfort while meeting the technical requirements of the Halcyon system. Patients in Group A were immobilized using a 3D‐printed headrest support mold in combination with a thermoplastic mask. The facial and cervical regions were further stabilized using a thermoplastic mask (Figure 1b). Group B was immobilized with a conventional headrest along with the same type of thermoplastic mask. The thermoplastic masks and headrests used in both groups were provided by Klarity Medical Products Co., Ltd. After completing immobilization, all patients underwent CT simulation using a large‐bore CT scanner (IntelliSpace IX/LX Workstations X, Philips). The scanning parameters were as follows: tube current of 200 mAs, tube voltage of 120 kV, slice thickness of 3 mm, and interslice spacing of 3 mm. The scan range extended from the top of the skull to the lower margin of the sternal notch. The acquired CT images were transmitted through the hospital's PACS system to the oncology radiotherapy management system for target contouring and radiotherapy planning by physicians and physicists. The reconstructed images were imported into the Halcyon treatment planning system (Eclipse 15.6, Varian, USA) for final target delineation and treatment planning.

### 2.4 Radiotherapy

All patients were positioned for treatment using the immobilization setup established during the pre‐treatment simulation. The treatment plan was retrieved, and image‐guided radiotherapy was initiated using cone beam computed tomography (CBCT) scanning. The scan range extended from the cranial vertex to the inferior border of the sternal notch. Standard scanning mode was used, with a slice thickness of 3 mm. A registration protocol was applied to account for variations in mobility across different cervical spine regions. Three regions of interest (ROIs)—the clivus^20^ C2–C4, and C5–C7 vertebral segments—were selected for automatic image registration between the CBCT and the simulation CT. This approach was used to determine the setup errors at the clivus, C4, and C7 vertebral levels.

### 2.5 Radiotherapy plan design

The same experienced medical physicist designed all radiotherapy plans in the study. The treatment planning system used was Eclipse version 15.6. The external air threshold was adjusted to reflect the CT value of the 3D‐printed material, ensuring that the actual CT value of the material was included in the dose calculation. This resulted in a treatment plan designated as Plan‐P (PLA material). All cases were planned using volumetric modulated arc therapy (VMAT). The entire plan, including parameters and settings, was then duplicated. The 3D‐printed material was contoured, and its CT value was set to −1000 to simulate the scenario without the presence of the 3D‐printed structure. The dose distribution was recalculated using the same optimization objectives and monitor units, resulting in a new plan referred to as Plan‐0.

### 2.6 Plan evaluation

The translational and rotational setup errors calculated from image guidance during each treatment session were compared between the two patient groups. According to the formula proposed by van Herk et al., the margin from the clinical target volume (CTV) to the planning target volume (PTV) is approximately calculated as:

Margin≈2.5∑+0.7σ, where ∑ represents the systematic setup error, and σ denotes the random setup error. Systematic error (Σ) was calculated as the mean displacement across all treatment fractions: $\sum =\frac{\sum_{i=1}^{n}\Delta_{i}}{n}\;$,

With Δ*i* representing the measured setup deviation (mm) in the *i*‐th treatment fraction, and n is the total number of fractions measured. Random error (σ) was derived as the standard deviation of residual displacements: $\sigma \;\;\;\;\;\;=\sqrt{\frac{\sum_{i=1}^{n}{\left(\Delta_{i}-\sum \right)}^{2}}{n}}$, where Δi is the measured setup deviation in the *i*‐th treatment fraction (mm), n represents the number of fractions, and ∑ represents the systematic error (mm).

The target volumes selected in the treatment plans included primary tumor volume (GTVnx), retropharyngeal lymph nodes (GTVrpn), cervical lymph nodes (GTVnd), planning gross tumor volume (PGTVnx), high‐risk clinical target volume (CTV1), planning high‐risk target volume (PTV1), prophylactic irradiation volume (CTV2), and planning prophylactic irradiation volume (PTV2). Organs at risk (OARs) evaluated included the lenses, optic chiasm, optic nerves, brainstem, and spinal cord. Target volume evaluation metrics included Mean dose (Dmean), Paddick Conformity Index (PCI), Homogeneity Index (HI), and Coverage Rate (CR).OARs evaluation metric:Maximum dose (Dmax);Mean dose (Dmean).The following formula obtains the Paddick Conformity Index (PCI):

$$\mathrm{PCI}\;=\frac{V_{\mathrm{ptv}\_\mathrm{ref}}}{V_{\mathrm{ptv}}}\times \frac{V_{\mathrm{ptv}\_\mathrm{ref}}}{V_{\mathrm{ref}}}\times 100\%,$$

Of which, Vptv is the volume of the prescription isodose line covering the PTV, Vptv is the volume of the PTV, and Vref is the total volume enclosed by the prescription isodose line. A PCI value of 1 indicates optimal conformity, that is, the prescribed dose fully conforms to the PTV. The following formula obtains the Homogeneity Index (HI):

$$\mathrm{HI}\;=\frac{D_{5}-D_{98}}{D_{50}}\times 100\%,$$

Of which D5 is the dose received by 5% of the PTV (near‐maximum dose), D98 is the dose received by 98% of the PTV (near‐minimum dose), and D50 is the median dose received by the PTV. An HI of 0 indicates ideal dose homogeneity within the target. The following formula obtains the Coverage Rate (CR):

$$\mathrm{CR}\;=\frac{V_{\mathrm{ptv}\_\mathrm{ref}}}{V_{\mathrm{ptv}}}\times 100\%,$$

This metric represents the proportion of the prescribed treatment volume (PTV) that is covered by the prescription dose.

### 2.7 Statistical method

All statistical analyses were performed using SPSS software version 22.0 (IBM Corp., Armonk, NY, USA). Categorical data were expressed as percentages, and continuous variables conforming to a normal distribution were presented as mean ± standard deviation (SD). Independent‐sample *t*‐tests were used to compare the differences between the two groups. A *p*‐value of less than 0.05 was considered statistically significant.

## 3 RESULTS

### 3.1 Setup error frequency of two positional fixation techniques

A total of 340 CBCT images were collected from 50 patients, including 164 scans in Group A and 176 scans in Group B. Among them, 7 CBCT scans in Group A and 12 in Group B required repeated imaging.

#### 3.1.1 Clivus region

In the left–right (LR) direction, Group A showed a distribution centered around 0 mm, with a peak frequency of 133 at 0 mm, indicating that most positioning errors were close to zero. In contrast, Group B had higher frequencies around −1 and 0 mm (80 and 126, respectively), with a more dispersed distribution, suggesting slightly poorer positioning stability.

**FIGURE 2:**
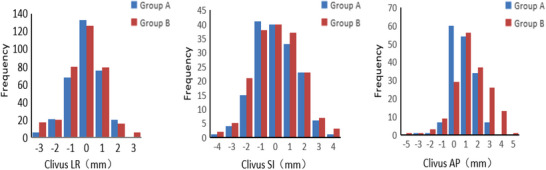
Setup error in the three‐dimensional direction of clivus area image‐guided registration.

In the superior–inferior (SI) direction, both groups had the highest frequency at 0 mm (40), but Group A demonstrated more variability, indicating greater susceptibility to error. In the anterior–posterior (AP) direction, Group A had the highest frequency at 0 mm (60), while Group B peaked at 1 mm (56). Additionally, Group A had fewer instances of large deviations, reflecting better positioning accuracy in this direction (Figure 2).

#### 3.1.2 C4 region

**FIGURE 3:**
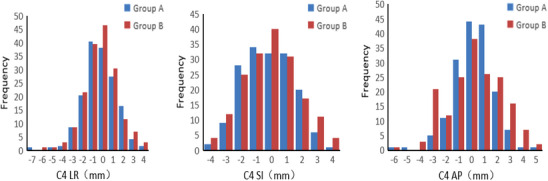
Setup error in the three‐dimensional direction of C4 cervical vertebra region image‐guided registration.

In the LR direction, both groups had high frequencies near 0 mm, with Group B showing a higher peak (46.5) compared to Group A (38). However, Group B also exhibited more frequent large deviations, indicating lower stability.In the SI direction, the peak frequency for Group A was at −1 mm (40.5), while Group B peaked at 0 mm (46.5), with overall similar distributions between the two groups. In the AP direction, both groups had higher frequencies near −1 and 0 mm, with comparable values (Group A: 34 and 32; Group B: 37 and 29), suggesting similar error distributions (Figure 3).

#### 3.1.3 C7 Region

**FIGURE 4:**
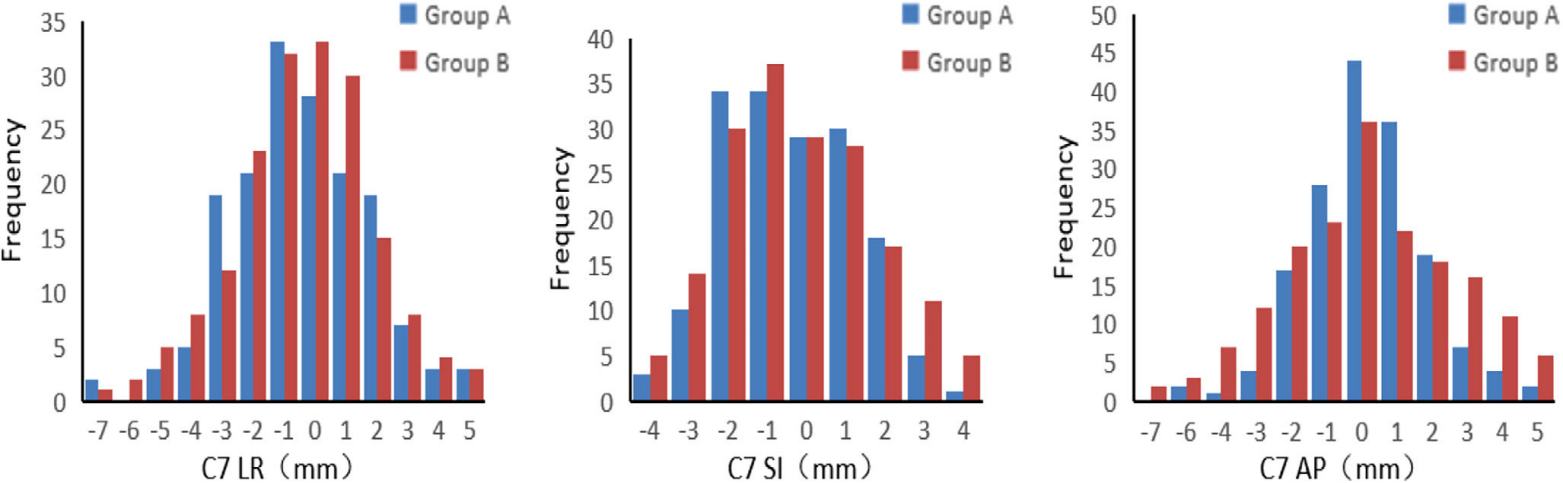
Setup error in the three‐dimensional direction of C7 cervical vertebra region image‐guided registration.

In the LR direction, both groups had peak frequencies around 0 mm (Group A: 28; Group B: 33), but Group B exhibited more instances of large deviations, implying slightly poorer stability. In the SI direction, both groups showed the highest frequency at 0 mm (33 each), with similar overall distributions. In the AP direction, Group A had the highest frequency at 0 mm (44), while Group B peaked at ‐1 mm (36). Fewer large deviations were observed in Group A, suggesting better accuracy in this direction (Figure 4).

#### 3.1.4 Overall stability comparison

Across all regions and directions, Group A demonstrated a more centralized error distribution, with fewer large deviations, near 0 mm, indicating superior overall positioning stability. In contrast, Group B exhibited more dispersed data and a higher frequency of large errors, suggesting relatively poorer stability.

#### 3.1.5 Regional stability differences

Group A showed a clear advantage in the AP direction of the clivus and C7 regions. In the LR direction of the C4 region, Group B exhibited noticeably poorer stability. In other regions and directions, the differences in positioning stability between the two groups were minimal.

### 3.2 Patient setup error and margin calculations

Statistically significant differences were observed in the anterior‐posterior (AP) direction among the three registration regions of interest (ROIs)—clivus, C4 vertebra, and C7 vertebra—across all 50 patients (Tables 1, 2, 3). In addition, statistically significant differences in roll rotational errors were found between groups for the C4 and C7 registration levels (Tables 2 and 3).

**TABLE 1:** Registration errors (mm) of CBCT images for 50 nasopharyngeal carcinoma radiotherapy patients using clivus as ROI.

Group	LR	SI	AP	Roll
Group A	0.8 ± 0.7	1.1 ± 0.9	1.1 ± 0.8	0.4 ± 0.4
Group B	0.8 ± 0.8	1.2 ± 0.9	1.4 ± 1.1	0.5 ± 0.5
*t*	0.474	1.457	2.506	0.412
*P*	0.636	0.146	0.013	0.681

**TABLE 2:** Registration errors (mm) of CBCT images for 50 nasopharyngeal carcinoma radiotherapy patients using C4 cervical vertebra level as ROI.

Group	LR	SI	AP	Roll
Group A	1.2 ± 1.0	1.4 ± 1.1	1.1 ± 1.0	0.5 ± 0.4
Group B	1.4 ± 1.1	1.4 ± 1.0	1.4 ± 1.3	0.6 ± 0.5
*t*	1.29	−0.445	2.325	2.042
*P*	0.198	0.656	0.021	0.041

**TABLE 3:** Registration errors (mm) of CBCT images for 50 nasopharyngeal carcinoma radiotherapy patients using C7 cervical vertebra level as ROI.

Group	LR	SI	AP	Roll
Group A	1.7 ± 1.3	1.4 ± 0.9	1.4 ± 1.3	0.3 ± 0.3
Group B	1.7 ± 1.4	1.5 ± 1.1	1.8 ± 1.5	0.4 ± 0.4
*t*	−0.336	1.112	2.274	2.469
*P*	0.737	0.267	0.024	0.014

Across all directions and regions, Group B consistently exhibited larger setup errors than Group A (Table 4). Notably, the planning target volume (PTV) margin expansions in Group B were significantly larger than those in Group A at the clivus level (LR, SI, and AP directions) and the C7 level (LR and AP directions). The most significant discrepancy was observed in the AP direction at the C7 level, with a margin of 4.65 mm in Group B versus 3.01 mm in Group A, representing a difference of 1.64 mm (Table 4).

**TABLE 4:** PTV external boundary (mm) calculated from systematic and random errors for nasopharyngeal carcinoma radiotherapy patients.

Registration site	Translation direction	Group A	Group B
Clivus	LR	1.71	2.23
	SI	2.52	3.15
	AP	1.97	3.11
C4	LR	2.90	2.83
	SI	2.87	3.90
	AP	2.36	4.42＊
C7	LR	2.91	4.31＊
	SI	2.97	3.93
	AP	3.01＊	4.65＊

*Note*: ^*^Indicates that the PTV expansion reached 3 mm, and the commonly assumed 3 mm external PTV boundary may not be sufficient to cover the target area.

Regarding directional differences, the superior‐inferior (SI) direction showed the most significant overall setup errors: at the clivus level, Group B had an SI deviation of 3.15 mm, while at the C4 level, the SI error reached 3.90 mm. The anterior–posterior (AP) direction demonstrated the most pronounced variability, with Group B requiring expansions exceeding 4 mm at both C4 and C7 levels (4.42 mm and 4.65 mm, respectively).

The C7 level showed the most significant overall errors, with Group B exceeding 4 mm in both LR and AP directions and even Group A reaching 3.01 mm in the AP direction. In contrast, the clivus level had the most minor errors, with Group A showing PTV margins <3 mm in all directions, and Group B shows a maximum of 3.11 mm in the AP direction.

### 3.3 PTVs dose and OAR dose differences

A comparison of two treatment plans, one with and one without the inclusion of 3D printing materials during dose calculation in the treatment planning system (TPS), revealed statistically significant differences in Dmean and PCI for the PTVnd + nx and PTV1 regions (*p* < 0.05). Additionally, significant differences were observed in Dmean, CR, and PCI for PTV2 (*p* < 0.05), as shown in Table 5.

**TABLE 5:** Dosimetric parameters of target area calculated by TPS.

Target area	Dose parameter	Plan‐P	Plan‐0	*t*	*p*
PTVnd+nx	Dmean/Gy	72.10 ± 1.00	72.71 ± 0.97	3.065	0.003
	CR/%	0.95 ± 0.04	0.97 ± 0.02	3.130	0.002
	PCI	0.82 ± 0.06	0.81 ± 0.07	−0.759	0.450
	HI	0.05 ± 0.02	0.05 ± 0.02	0	1
PTV1	Dmean/Gy	68.73 ± 0.80	69.10 ± 0.77	2.333	0.022
	CR/%	0.97 ± 0.01	0.98 ± 0.01	4.950	<0.001
	PCI	0.48 ± 0.09	0.48 ± 0.09	0	1
PTV2	Dmean/Gy	61.87 ± 1.05	62.54 ± 1.03	3.189	0.002
	CR/%	0.91 ± 0.05	0.95 ± 0.05	3.960	<0.001
	PCI	0.84 ± 0.04	0.87 ± 0.02	4.696	<0.001

In the dosimetric evaluation of organs‐at‐risk (OARs) adjacent to the target volume, the maximum doses to the brainstem, spinal cord, left lens, left optic nerve, and right optic nerve were significantly higher in the Plan‐p group compared with the Plan‐0 group (*p* < 0.05). However, no statistically significant differences were observed between the two plans in terms of the mean doses to these OARs (*p* > 0.05; Tables 6 and 7).

**TABLE 6:** Comparison of maximum dose to critical organs calculated by TPS (Gy).

Group	Brainstem	Spinal cord	Left lens	Right lens	Left optic nerve	Right optic nerve
Plan‐P	48.36 ± 7.38	29.92 ± 1.53	5.67 ± 0.95	5.48 ± 0.93	41.04 ± 13.82	43.57 ± 15.10
Plan‐0	43.96 ± 6.87	28.67 ± 1.46	5.16 ± 0.78	5.37 ± 0.91	31.29 ± 10.54	31.14 ± 11.32
*t*	−2.182	−2.955	−2.075	−0.423	−2.805	−3.293
*p*	0.034	0.004	0.043	0.674	0.007	0.002

**TABLE 7:** Comparison of mean dose to critical organs calculated by TPS (Gy).

Group	Brainstem	Spinal cord	Left lens	Right lens	Left optic nerve	Right optic nerve
Plan‐P	20.49 ± 4.87	15.88 ± 2.57	4.18 ± 1.54	4.13 ± 1.85	30.47 ± 3.91	31.89 ± 4.73
Plan‐0	20.42 ± 4.79	15.83 ± 2.51	4.16 ± 1.33	4.09 ± 1.72	30.43 ± 3.90	31.84 ± 4.70
*t*	−0.072	−0.097	−0.049	−0.079	−0.051	−0.053
*p*	0.943	0.923	0.961	0.937	0.960	0.958

## 4 DISCUSSION

The comparison between the application of 3D‐printed headrest support mold devices and traditional fixation devices in the radiotherapy of nasopharyngeal carcinoma revealed that the positioning errors in the 3D‐printed support group were significantly lower in multiple directions compared to the traditional group. Several studies support the advantage of personalized positioning technologies in reducing setup errors. For example, Guo W et al.^21^ demonstrated that the use of 3D‐printed individualized head molds significantly improved the X‐axis rotational setup error, with a trend towards reduced translation and rotational setup errors in other directions. Additionally, Ma et al.^22^ demonstrated that the use of 3D‐printed oral splints effectively reduces setup errors, thereby improving the accuracy of radiotherapy. Furthermore, MRI equipment with analytical capabilities for measuring related volumes of motion has demonstrated lower absolute 3D errors when using a 3D‐printed fixation mask, with higher fixation precision per session compared to a traditional stereotactic high‐precision mask.^23^

The results of this study indicate significant differences in setup errors between the two groups when registering regions of interest (ROIs) at the Clivus, C4, and C7 levels in the AP direction. Additionally, significant differences were observed in Roll rotation errors when registering ROIs at C4 and C7. The 3D‐printed headrest support mold group (Group A) exhibited more minor setup errors in specific directions. This can be attributed to the personalized customization capabilities of 3D printing technology. The 3D‐printed head and neck support mold is generated based on the patient's own simulation CT images, converted into 3D models, and then printed, providing a better fit to the patient's body contours and resulting in more stable and accurate support. In contrast, traditional methods, such as the use of headrests and thermoplastic masks, face challenges due to the inability of the headrest to be precisely adjusted to individual patient differences. This inconsistency in patient positioning results in larger setup errors during the fixation process.

From the perspective of radiotherapy precision, minimizing setup errors is crucial for enhancing the therapeutic efficacy of treatment. Cognitive impairments following radical intensity‐modulated radiation therapy (IMRT) for nasopharyngeal carcinoma are common and are associated with radiation doses to the entire brain, hippocampus, and temporal lobes^24^ Due to the complex anatomical structures in these regions, significant positioning errors may result in deviations in the radiation dose from the tumor target area. This not only compromises tumor control but also increases the risk of radiation‐induced damage to normal tissues. For example, excessive radiation to the brainstem can cause severe neurological complications, adversely affecting the patient's quality of life and prognosis^25^ Therefore, the advantages of 3D‐printed personalized positioning techniques in reducing setup errors play a significant role in enhancing the accuracy and safety of nasopharyngeal carcinoma radiotherapy. The calculated results of the PTV (Planning Target Volume) margin show that there are differences between Group A and Group B in multiple directions at different cervical vertebral levels, with some regions having a PTV margin extension of 3 mm or more. This suggests that the traditionally accepted 3 mm margin may be insufficient to cover the target area fully. The 3D‐printed personalized positioning technique more accurately reflects the patient's actual positional changes, and its more minor positioning errors enable a more precise determination of the PTV boundary. For Group A patients, the well‐fitting 3D‐printed headrest support molds and thermoplastic masks reduce positional shifts during treatment. As a result, both system errors and random errors are more minor, leading to a relatively narrower PTV margin. In contrast, Group B, due to the limitations of traditional fixation methods, experiences larger positional movements, necessitating a broader planning target volume (PTV) margin to ensure complete coverage of the tumor target area.

Accurate determination of the PTV (Planning Target Volume) margin is of significant clinical importance for optimizing radiotherapy planning. On the one hand, minimizing positioning errors while ensuring tumor control helps reduce the irradiated volume of normal tissues and lowers the risk of radiation‐induced complications. Ding Z. et al.^26^ introduced the first five setup errors into a VMAT plan. They recalculated the doses, observing that the nearby OARs (Organs at Risk) experienced significant dose variations due to setup errors. The average ΔDmax for the brainstem and spinal cord reached 1.85 Gy and 1.51 Gy, respectively, while the average ΔNTCP for the bilateral parotid glands increased by 6.17% (left) and 7.70% (right). Larger setup errors dramatically increased the risk of parotid dysfunction. On the other hand, the application of 3D‐printed headrests has demonstrated smaller PTV margins, allowing radiotherapy doses to be more effectively concentrated on the tumor target volume, thereby enhancing local tumor control rates. Cai et al.^27^ found that when setup errors were incorporated into each treatment fraction, and doses were recalculated, larger setup errors decreased the uniformity and consistency of dose distribution in the treatment plan, thereby lowering the tumor's local control rate. More minor setup errors play a positive role in improving the therapeutic outcomes of nasopharyngeal carcinoma radiotherapy and enhancing patient prognosis.

3D printing enables the rapid design and fabrication of individualized immobilization devices and has been widely applied in clinical settings to improve patient positioning accuracy^28^, ^29^ However, studies focusing on the dosimetric impact of 3D‐printed head and neck immobilization materials in radiotherapy remain relatively limited. Hsu EJ et al.^30^ investigated the use of 3D‐printed headrests for treating tumors located on the scalp and neck, demonstrating that such devices can significantly reduce air gaps between the head and the headrest—this enhancement in setup reproducibility results in a more uniform dose distribution for superficial tumors. Yin et al.^31^ evaluated the dosimetric effects of 3D‐printed immobilization devices in head and neck radiotherapy, showing that the inclusion of a 3D‐printed headrest reduced target volume V100% and Dmean while increasing the Dmax to the brainstem and optic nerves. In addition, Bustillo et al.^32^ developed customizable tissue‐equivalent phantoms using 3D printing, reporting point dose discrepancies of less than 2% and an overall gamma pass rate of more than 90%, indicating an acceptable agreement with treatment planning systems (TPS) and confirming the accuracy of additive manufacturing techniques and materials in radiotherapy applications. In the present study, personalized immobilization devices for radiotherapy of nasopharyngeal carcinoma were fabricated using PLA‐based 3D printing technology. Dosimetric analysis within the TPS revealed that the presence of 3D‐printed materials can affect the dose distribution to the target volume, particularly in terms of mean dose and coverage rate. The dosimetric analysis indicated that the presence of 3D‐printed materials can influence the dose distribution within the target volume, particularly with respect to mean dose and coverage. At the same time, careful attention must be directed to the potential increase in the maximum dose to adjacent organs at risk (OARs) associated with the use of 3D‐printed materials. Such considerations are critical to ensuring both clinical safety and treatment accuracy.

In conventional radiotherapy, it is generally assumed that the choice of patient immobilization materials has minimal impact on treatment planning, and therefore, the influence of immobilization devices on radiotherapy is often overlooked. However, in practice, common materials such as foam cushions or water‐solidified plastic headrests can significantly affect the dose distribution to the target area. For 3D‐printed immobilization devices, factors such as thickness, internal infill ratio, and infill pattern may alter the radiation dose delivered during treatment.^33^, ^34^, ^35^, ^36^ Therefore, it is critical to determine whether novel materials like 3D‐printed supports can be reasonably approximated as equivalent to air in dose calculations, or whether their potential dosimetric effects must be explicitly considered. In this study, the thickness and infill ratio of the 3D‐printed headrest were strictly controlled, and sub‐millimeter printing precision was employed to minimize dose deviations associated with manufacturing inaccuracies. The results demonstrate that 3D‐printed materials indeed affect the dose delivered to the target volume and may lead to steeper dose gradients for adjacent organs at risk (OARs). These findings highlight the importance of further evaluating the dosimetric effects of 3D‐printed materials, particularly in the context of patient‐specific immobilization design, to ensure accurate and safe radiotherapy delivery.

The PLA material used in this study has been applied clinically for many years and has been consistently demonstrated to be both biocompatible and safe.^37^, ^38^ According to the International Organization for Standardization (ISO) 10993 guidelines for the biological evaluation of medical devices.^39^ PLA exhibits negligible cytotoxicity and low hemolytic activity.^40^, ^41^, ^42^, ^43^ Furthermore, its degradation product, lactic acid, is a naturally occurring metabolite, further supporting its safety profile in clinical applications.^44^ These properties render PLA suitable for skin contact and short‐term, non‐invasive medical use. As a semi‐crystalline aliphatic polyester, PLA primarily undergoes chain scission and crosslinking when exposed to radiation.^45^, ^46^, ^47^ The radiation doses received by PLA headrests during standard nasopharyngeal carcinoma IMRT are relatively low, making the likelihood of radiation‐induced material failure during treatment minimal.^48^ Throughout the entire treatment course, visual and functional inspections of the printed PLA headrests revealed no evidence of warping, cracking, or deformation. These findings indicate that PLA headrests maintain structural stability under routine radiotherapy conditions and are well‐suited for use in 3D‐printed immobilization devices for external beam therapy. Nevertheless, given the relatively small sample size of this study, further research is warranted to comprehensively evaluate the biocompatibility and material stability of 3D‐printed PLA to ensure that no potential adverse effects arise during radiotherapy.

Although 3D‐printed personalized immobilization technology demonstrated clear advantages in this study, several challenges remain for its broader clinical implementation. First, the initial investment in 3D printing equipment and materials is relatively high, which may limit its application in regions with limited medical resources. Second, the fabrication of 3D‐printed immobilization devices requires skilled personnel and specialized software support, thereby imposing higher technical demands on medical institutions. Furthermore, the biocompatibility and material stability of current 3D printing materials still require further investigation and validation to ensure that no potential adverse effects occur during the radiotherapy process.

## 5 CONCLUSION

This study demonstrates that 3D‐printed personalized immobilization technology provides a high‐precision and highly adaptable solution for radiotherapy in patients with nasopharyngeal carcinoma. Its clinical application can effectively reduce setup errors and allow for narrower PTV margins. The incorporation of 3D‐printed materials exerts a measurable influence on target volume dose distribution and may notably increase the maximum dose delivered to adjacent critical organs.

## AUTHOR CONTRIBUTIONS

Study conception and design: Zeyu Ding, Guo‐quan Li, Pingjing Cheng. Administrative support: Jun Han. Acquisition of data: Zeyu Ding, Guo‐quan Li, Qi Liu, Xiaohui Zhu, and Ye Wang. Analysis and interpretation of data: Guo‐quan Li, Jun Kang, Zhiyong Yang, and Jinghua Ren. Drafting of the manuscript: Zeyu Ding, Guo‐quan Li, and Pingjing Cheng. Final approval of manuscript: All authors.

## CONFLICT OF INTEREST STATEMENT

The authors declare no conflicts of interest.

## ETHICS STATEMENT

Our study was approved by the Ethics Committee of Union Hospital of Huazhong University of Science and Technology (No: [2023] IEC(0738).
